# Clinical and laboratory considerations: determining an antibody-based composite correlate of risk for reinfection with SARS-CoV-2 or severe COVID-19

**DOI:** 10.3389/fpubh.2023.1290402

**Published:** 2023-12-28

**Authors:** Stefan Holdenrieder, Carlos Eduardo Dos Santos Ferreira, Jacques Izopet, Elitza S. Theel, Andreas Wieser

**Affiliations:** ^1^Institute of Laboratory Medicine, German Heart Centre Munich, Technical University Munich, Munich, Germany; ^2^Clinical Laboratory, Department of Laboratory Medicine, Hospital Israelita Albert Einstein, São Paulo, Brazil; ^3^Laboratory of Virology, Toulouse University Hospital and INFINITY Toulouse Institute for Infections and Inflammatory Diseases, INSERM UMR 1291 CNRS UMR 5051, University Toulouse III, Toulouse, France; ^4^Division of Clinical Microbiology, Department of Laboratory Medicine and Pathology, Mayo Clinic, Rochester, MN, United States; ^5^Division of Infectious Diseases and Tropical Medicine, University Hospital, LMU Munich, Munich, Germany; ^6^German Centre for Infection Research (DZIF), Munich, Germany; ^7^Faculty of Medicine, Max Von Pettenkofer Institute, LMU Munich, Munich, Germany; ^8^Immunology, Infection and Pandemic Research, Fraunhofer Institute for Translational Medicine and Pharmacology ITMP, Munich, Germany

**Keywords:** SARS-CoV-2, immunity, antibodies, models and modeling, innate and adaptive immune response, patient-centered care, vaccines, clinical utility

## Abstract

Much of the global population now has some level of adaptive immunity to SARS-CoV-2 induced by exposure to the virus (natural infection), vaccination, or a combination of both (hybrid immunity). Key questions that subsequently arise relate to the duration and the level of protection an individual might expect based on their infection and vaccination history. A multi-component composite correlate of risk (CoR) could inform individuals and stakeholders about protection and aid decision making. This perspective evaluates the various elements that need to be accommodated in the development of an antibody-based composite CoR for reinfection with SARS-CoV-2 or development of severe COVID-19, including variation in exposure dose, transmission route, viral genetic variation, patient factors, and vaccination status. We provide an overview of antibody dynamics to aid exploration of the specifics of SARS-CoV-2 antibody testing. We further discuss anti-SARS-CoV-2 immunoassays, sample matrices, testing formats, frequency of sampling and the optimal time point for such sampling. While the development of a composite CoR is challenging, we provide our recommendations for each of these key areas and highlight areas that require further work to be undertaken.

## Introduction

The COVID-19 pandemic, caused by severe acute respiratory coronavirus 2 (SARS-CoV-2) led to unprecedented, accelerated vaccine development (1) and expansive roll-out programs (2, 3). Much of the global population now has some level of adaptive immunity to SARS-CoV-2 induced by exposure to the virus (natural infection), vaccination, or a combination of both (hybrid immunity).

Natural infection induced by, and/or vaccination against, SARS-CoV-2 leads to the development of both binding and neutralizing antibodies (nAbs) (4, 5), and the induction of T-cell responses during active immune reaction and clearance of infection (6). Key questions that subsequently arise relate to the duration and the level of protection an individual might expect based on their infection and vaccination history. Studies of those infected early in the pandemic documented that natural SARS-CoV-2 infection afforded some level of protection against reinfection in most individuals, and that subsequent reinfections were typically less severe than the primary episode (Table 1). However, SARS-CoV-2 has high rates of mutation and heavily mutated variants have emerged (21). Most significant are the “variants of concern” (VOCs) (22), and there is now ample evidence that protection against reinfection with the B.1.1.529/21 K (Omicron) variant (23, 24) is dramatically reduced compared with previous variants (Table 1).

**Table 1:** Selection of peer-reviewed publications assessing reinfection or risk of severe COVID-19 after natural infection (ordered by study end date, earliest to most recent).

	Study			Outcome measures of protection or risk		
	Total size (enrolled; before exclusions)	Time period	Reported lineage	Reported outcome measure (protection, risk, reinfection rate)	Repeat infection outcome (selected comparisons, terminology as reported)	Severe COVID-19 outcome (selected comparisons, terminology as reported)
Primary publications						
Hansen et al. (7) Non-vaccinated individuals Denmark	~ 4 million individuals	February 26, 2020–December 31, 2020	None	Protection	Protection against repeat infection in those^1^ < 65 years: 80.5% (95% CI: 75.4–84.5) ≥ 65 years: 47.1% (96% CI 24.7–62.8)	Not assessed
Abu-Raddad et al. (8) Non-vaccinated individuals^2^ Qatar	192,984 individuals	April 16, 2020–December 31, 2020	None	Protection	Efficacy of natural infection against reinfection^3^ 95.2% (95% CI: 94.1–96.0)	Not assessed Of 129 cases with good or some evidence of reinfection, one reinfection was severe, two were moderate, and none were critical or fatal
Hall et al. (9) Non-vaccinated and vaccinated individuals UK	30,625 individuals	June 18, 2020–January 11, 2021	Not specified B.1.1.7	Risk	Risk of reinfection causing^4^ COVID-19 symptoms: aIRR 0.074 (95% CI: 0.06–0.10) All events (COVID-19 symptoms, other symptoms, asymptomatic): aIRR 0.159 (95% CI: 0.13–0.19)	Not assessed
Lumley et al. (10) Non-vaccinated and vaccinated individuals UK	13,109 individuals	March 27, 2020–February 28, 2021	Non-S-gene target failure B.1.1.7	Risk	Risk of PCR-positive result (symptomatic or asymptomatic) in Unvaccinated seropositive^5^: aIRR 0.02 (95% CI: 0.01–0.18)	Not assessed
Abu-Raddad et al. (11) Non-vaccinated and vaccinated individuals Qatar	193,233 individuals	Before November 1, 2020–March 3, 2021	B.1.1.7 Variants of unknown status	Protection	Efficacy of natural infection against reinfection with^6^ B.1.1.7, prior PCR-confirmed infection: 97.5% (95% CI: 95.7–98.6) B.1.1.7, prior antibody-positive result: 97.0% (95% CI: 92.5–98.7) Unknown variant, prior PCR-confirmed infection: 92.2% (95% CI: 90.6–93.5) Unknown variant, prior antibody-positive result: 94.2% (95% CI: 91.8–96.0)	Not assessed
Chemaitelly, et al. (12) Unvaccinated individualsQatar	380,914 individuals	Before January 1, 2021–April 21, 2021^7^	B.1.351 B.1.1.7 Variants of unknown status	Protection	Efficacy of natural infection against reinfection with^8^ B.1.351: 92.3% (95% CI: 90.3–93.8) B.1.1.7: 97.6% (95% CI: 95.7–98.7) Variants of unknown status: 87.9% (95% CI: 84.7–90.5)	Not assessed
Nordström et al. (13) Non-vaccinated and vaccinated individuals Sweden	~3.5 million individuals (3 cohorts)	March 20, 2020–September 5, 2021	Alpha B.1.1.7 Beta B.1.351 Gamma P.1 Delta B.1.617.2	Risk	Risk of reinfection in those with Natural immunity^9^: aHR 0.05 (95% CI: 0.05–0.05) One-dose hybrid immunity^10^: aHR 0.42 (95% CI: 0.38–0.47) One-dose hybrid immunity^11^: aHR 0.55 (95% CI: 0.39–0.76) Two-dose hybrid immunity, overall^12^: aHR 0.34 (95% CI: 0.31–0.39)	Risk of hospitalization (HR) Two-dose hybrid immunity^13^: 0.10 (95% CI: 0.04–0.22)
Altarawneh et al. (14) Non-vaccinated and vaccinated individuals Qatar	~2.3 million individuals	March 23, 2021–November 18, 2021	Alpha Beta Delta Omicron	Protection	Effectiveness of previous infection in preventing reinfection with^14^ Alpha: 90.2% (95% CI: 60.2–97.6) Beta: 85.7% (95% CI: 75.8–91.7) Delta: 92.0% (95% CI: 87.9–94.7) Omicron: 56.0% (95% CI: 50.6–60.9)	Effectiveness of previous infection in preventing severe, critical, or fatal disease caused by Alpha: 69.4% (95% CI: −143.6 to 96.2) Beta: 88.0% (95% CI: 50.7–97.1) Delta: 100% (95% CI: 43.3–100) Omicron: 87.8% (95% CI: 47.5–97.1)
Pulliam et al. (15) Non-vaccinated and vaccinated individuals South Africa	~2.9 million individuals	March 4, 2020–January 31, 2022	Beta (B.1.351) Delta (B.1.617.2) Omicron (B.1.1.529)^15^	Risk	Risk of reinfection during^16^ Wave 2 (Beta-driven) versus Wave 1: relative HR 0.71 (95% CI: 0.60–0.85) Wave 3 (Delta-driven) versus Wave 1: relative HR 0.54 (95% CI: 0.45–0.64) Wave 4 (Omicron-driven) versus Wave 1: relative 1.70 (95% CI: 1.44–2.04)	Not assessed
Guedes et al. (16) Non-vaccinated and vaccinated individuals Brazil	25,750 real-time RT-PCR tests performed	March 10, 2020–March 20, 2022	Pre-VOC Gamma Delta Omicron	Reinfection rate	Reinfection rate during the Omicron variant period^17^: Before 0.8% vs. after 4.3%; *p* < 0.001	Not assessed 281/281 reinfections were mild
Chemaitelly et al. (17) Unvaccinated individuals Qatar	Up to 3.3 million individuals	February 28, 2020– June 5, 2022^18^	Pre-Omicron (ancestral, Alpha, Beta, Delta) Omicron (BA.1, BA.2, BA.4, BA.5)	Protection	Effectiveness of pre-Omicron primary infection^19^ Against pre-Omicron reinfection: 85.5% (95% CI: 84.8–86.2%) Effectiveness peaked at 90.5% (95% CI: 88.4–92.3%) in the 7th month after the primary infection, waning to ~70% by the 16th month Against Omicron reinfection: 38.1% (95% CI: 36.3–39.8%), declining with time since primary infection	Effectiveness of pre-Omicron primary infection^20^ Against severe, critical, or fatal COVID-19 due to Omicron reinfection: 88.6% (95% CI: 70.9–95.5) Against severe, critical, or fatal COVID-19 reinfection (irrespective of the variant of primary infection or reinfection): 97.3% (95% CI: 94.9–98.6)
Bowe et al. (18) Non-vaccinated and vaccinated individuals USA	~ 5.8 million individuals	March 1, 2020–June 25, 2022	Pre-Delta Delta Omicron	Risk	Not assessed	Risk of all-cause mortality (HR)^21^ 2.17 (95% CI: 1.93–2.45) Risk of hospitalization (HR) 3.32 (95% CI: 3.13–3.51)
Yang et al. (19) Non-vaccinated and vaccinated individuals Malaysia	482 individuals	January 31, 2022–July 31, 2022^22^	Non-Omicron Omicron	Risk	Risk of reinfection in those with Pre-Omicron natural infection^23^: aHR 0.41 (95% CI: 0.27–0.62)	Not assessed
Meta-analyses						
Stein et al. (20) Global systematic review and meta-analysis of 65 studies from 19 countries	Various	Up to September 31, 2022	Ancestral Mixed Alpha (B.1.1.7) Beta (B.1.351) Delta (B.1.617.2) Omicron BA.1 variants	Protection	Pooled estimate of protection from past infection (with various variants) against reinfection with Ancestral: 84.9 (95% UI 72.8–91.8) Alpha: 90.0% (95% UI 54.8–98.4) Beta: 85.7% (95% UI 83.4–87.7) Delta: 82.0 (95% UI 63.5–91.9) Omicron BA.1: 45.3% (95% UI 17.3–76.1)	Pooled estimate of protection against severe disease caused by Ancestral: 78.1% (95% UI 34.4–96.5) Alpha: 79.6% (95% UI 43.3–95.3) Beta: 88% (95% UI 50.7–97.1)^24^ Delta: 97.2% (95% UI 85.2–99.6) Omicron BA.1: 81.9% (95% UI 73.8–88.0)

^1^Derived as 1− adjusted relative risk. The rates of infection during the second surge were compared across those with a positive or negative PCR test from the first surge. The rate of infection was calculated as the number of individuals with positive PCR tests during the second surge divided by the cumulative number of person-days at risk. ^2^Qatar launched its vaccination campaign on December 21, 2020, around the time this study was concluded (December 31, 2020), so very few individuals had been vaccinated at time of this study. ^3^Derived as 1− the ratio of the incidence rate of reinfection in the antibody-positive cohort to the incidence rate of infection in the antibody-negative cohort. ^4^Derived as 1− adjusted incident rate ratio. ^5^Compared incidence in each follow-up group to unvaccinated seronegative healthcare workers. ^6^Derived as 1− the ratio of the incidence rate of reinfection in the PCR-confirmed (or antibody-positive) cohort to the incidence rate of infection in the antibody-negative cohort. ^7^This timeframe coincided with the beginning of the decline of the B.1.1.7 wave and the rapid expansion of the B.1.351 wave that peaked early April 2021. ^8^Derived as 1− the ratio of the incidence rate of reinfection in the cohort of individuals with a prior PCR-confirmed infection to the incidence rate of infection in the antibody-negative cohort. ^9^Calculated vs. no immunity and after 3 months of follow-up. ^10^Calculated vs. natural immunity and during the first 2 months of follow-up. ^11^Calculated vs. natural immunity and after 2 months of follow-up. ^12^Calculated vs. natural immunity. ^13^Calculated vs. natural immunity. ^14^Derived as 1− odds ratio of prior infection in cases (PCR-positive persons with variant infection) vs. controls (PCR-negative persons). ^15^Period of Omicron emergence: November 1, 2021 to November 30, 2021. ^16^Estimated relative hazard ratios for reinfection during specified wave versus primary infection during the first wave. ^17^Calculated as number of reinfection cases before and after the Omicron variant considering the total accumulated number of SARS-CoV-2 infections in both periods. ^18^Three individual studies (pre-Omicron reinfection, Omicron reinfection, COVID-19 severity reinfection) spanning different time periods. ^19^Derived as 1– adjusted hazard ratio, where the hazard ratio compared incidence of infection in both cohorts. Incidence rate of infection in each cohort defined as the number of identified infections divided by the number of person-weeks contributed by all individuals in the cohort. ^20^Cox regression analysis. Severity, criticality, and fatality defined as per WHO guidelines. ^21^Calculated for reinfection vs. no reinfection. ^22^The Omicron-dominant period in Malaysia was estimated to start from early February 2022. ^23^Calculated vs. Omicron-dominant period. ^24^Single study. aRR, adjusted risk ratio; aIRR, adjusted incidence risk ratio; aHR, adjusted hazard ratio; CI, confidence interval; HR, hazard ratio; OR, odds ratio; PE_S_, effectiveness of prior infection in preventing reinfection; real-time RT-PCR, real-time reverse transcription polymerase chain reaction; UI, uncertainty interval.

Any descriptor of immunity based on patient history will encompass a population of individuals with vastly variable exposure to vaccines and viral variants with differing orders of immune challenge intensity. Unrecognized “silent infections,” especially in Omicron-positive subjects with underlying immunity, further complicate the assessment. Therefore derivation of potential immunity based on patient history requires assistance from a surrogate composite score to inform about protection and to aid decision making.

### Correlates of protection or risk

In vaccinology, a correlate of protection (CoP) reflects a statistical non-causal relationship between an immune marker and protection after vaccination (25). Most accepted CoPs are based on antibody measurements (26) and vary depending on the clinical endpoint, for example protection from (symptomatic) infection or severe disease. In contrast, a correlate of risk (CoR) can be used as a measurement of an immunologic parameter that is correlated with a study endpoint (27) and can predict a clinical endpoint in a specified population with a defined future timeframe. Notably, antibody markers have been used as correlates of immune function in clinical trials of SARS-CoV-2 vaccine efficacy (VE) (28–33), and for identifying the risk of symptomatic infection by VOCs (34, 35). In VE trials, a CoR can be a CoP if the CoR reliably predicts VE against the clinical endpoint, thereby acting not just as an intrinsic susceptibility factor or marker of pathogen exposure. In this case, the CoR could be a surrogate of the endpoint and could be useful for licensure of new vaccines.

A CoR would likely comprise a measure of the immune component plus determinants that act to modify such a measure (a multi-component composite CoR). While there is no scientific evidence for an absolute humoral or cellular CoP against SARS-CoV-2, identification of a multi-component composite CoR might be useful to guide the use of vaccines or patient management. In general, the immune component of a composite CoR should be easily measured by widely available technologies that are amenable to automation, are scalable, cost-efficient, and have a rapid turn-around time. Given the relative complexity, cost and pre-analytic requirements for cellular immune response testing, the preferred candidate for the immune component of a CoR would be detection of humoral immune response(s) (i.e., antibody). This perspective evaluates the various elements that need to be accommodated in the development of an antibody-based composite CoR for reinfection with SARS-CoV-2 or severe COVID-19.

## A composite CoR: a brief summary of extrinsic viral and intrinsic host elements that should be considered

### Variation in exposure dose and transmission route

Viral load varies widely between infected individuals and over time (36), with viral emissions independent of symptom severity (37). Exposure to SARS-CoV-2 is tempered by the use of personal protective measures and, at the population level, adherence to public health measures that reduce exposure has been variable (38, 39), making assessment of exposure dose complex.

Controlled human infections to directly study the impact of viral inoculum and disease severity are controversial (40), and only one human challenge trial of SARS-CoV-2 using a single low inoculum dose has been reported to date (41). However, the initial infective dose of SARS-CoV-2 is thought to be associated with disease severity (42–44), since relationships between dose and severity exist for many other viral infections (44). Evidence from SARS-CoV-2 animal models suggests that the route of transmission similarly affects disease severity (45).

### Viral genetic variation

Risk reduction depends on the dominant variant in circulation. Continued evolution of SARS-CoV-2 can lead to significant changes in viral transmission and impact reinfection rates (46). Mechanistically, the receptor binding domain (RBD) within the viral spike (S) glycoprotein engages in initiation of infection via interaction with the angiotensin converting enzyme-2 (ACE2) receptor (47). The RBD is a target for many nAbs (47) and mutations are frequently located at the RBD–ACE2 interface (48). It is therefore not surprising that changes to the viral epitope can reduce antibody binding (48), helping to drive immune escape from anti-RBD nAbs (49), decreasing previously generated protective immunity (50–52), and leading to variant-specific risks of severe illness (53, 54).

### Patient factors

Patient differences impact susceptibility to reinfection and disease severity. The immune response declines with increasing age (55, 56), and age is the strongest predictor of SARS-CoV-2 infection–fatality ratio (57). Older individuals have been shown to exhibit reduced binding antibody titers and neutralization following vaccination (58–60). Pregnant women are also at high risk of severe outcomes (61). Similarly, immunocompromised or immunosuppressed individuals, or those affected by cancer or human immunodeficiency virus (HIV), exhibit reduced immune responses to infection or an increased risk of hospitalization (62–66). Other co-morbidities are frequently observed in those with severe COVID-19 (67, 68).

### Vaccination status and exposure history

COVID-19 vaccines include recombinant subunit, nucleic acid, viral vector and whole virus vaccines, among others, and some vaccines have been adapted for Omicron variants (69). The use of different vaccines, combinations, the number of boosters received, the interval between boosters, the occurrence of natural infection, and combinations thereof, trigger the immune system to varying degrees in depth, breadth or duration of response (35, 66, 70–83). Pre-existing heterotypic immunity, due to past infections with other coronaviruses, may also influence the immune response to SARS-CoV-2 (84, 85).

Following primary infection, severely ill patients exhibit higher binding and neutralizing antibody titers or activity compared with individuals with mild disease (86–91). Persistence of nAbs has also been associated with disease severity (92). In the event of reinfection, there is an implicit assumption that nAb titers ameliorate severe COVID-19 (93, 94). In brief, in infection-naïve individuals, post-vaccination antibody titers (anti-S IgG and nAbs) correlate with higher vaccine efficacy (71), and post-vaccination anti-RBD IgG and nAbs levels associate with protection against infection and symptomatic disease even during the Omicron era (95) or inversely correlate with risk of death (anti-S IgG below 20th percentile) (96). Generally, individuals with higher nAbs (levels or capacity) are considered increasingly protected from infection (97–99), symptomatic reinfection (99–101), severe disease (100), or death (102) compared with individuals with lower nAbs. There is evidence that neutralization capacity can be strain specific (103).

**Figure 1:**
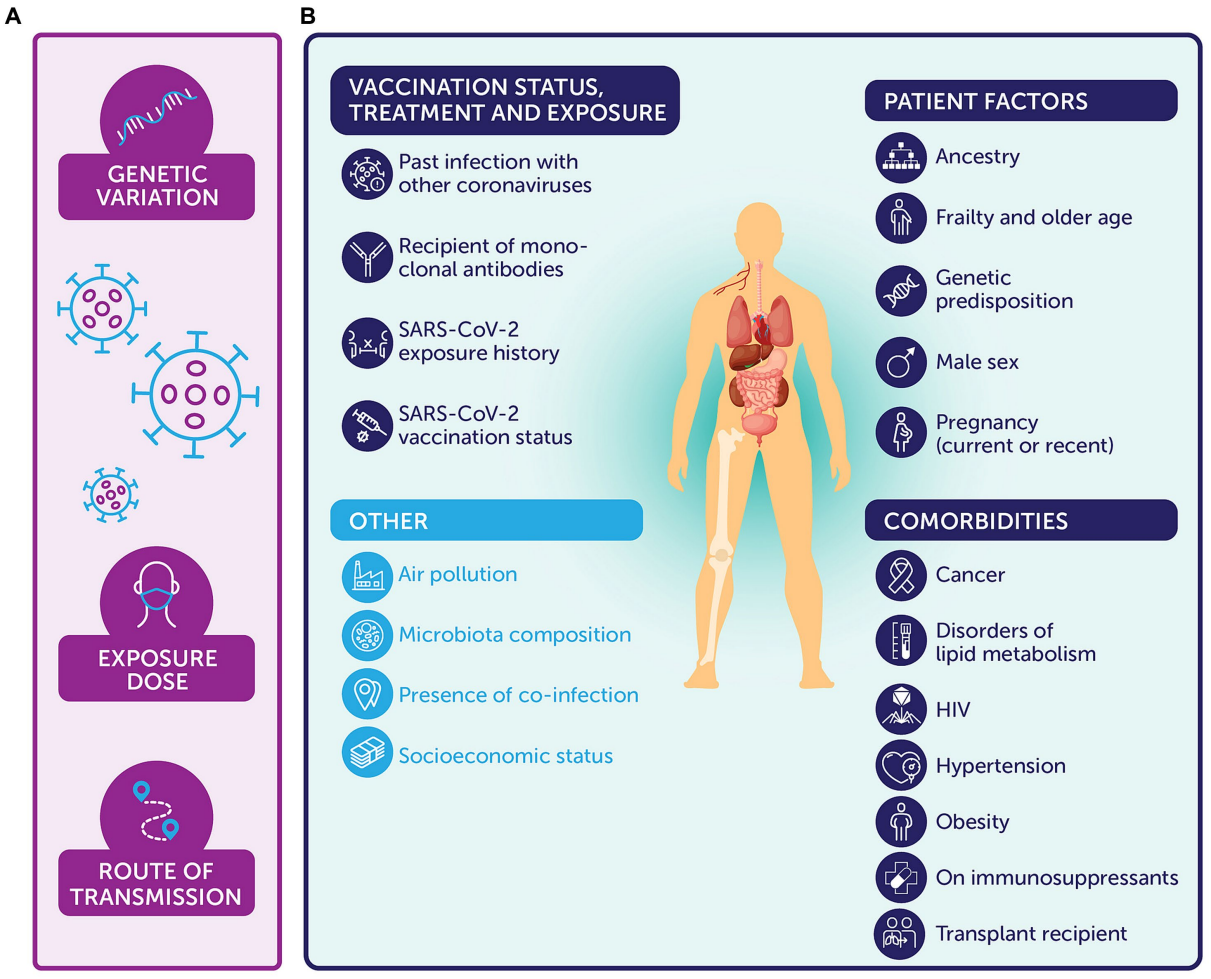
Summary of host and viral elements that can impact the immune response and response to SARS-CoV-2 (selected examples, not exhaustive, variables ordered alphabetically within figure [not according to importance]). **(A)** Viral factors include genetic variation (50–54), the exposure dose (42–44), and route of transmission (45). **(B)** Host factors include patient factors, such as: ancestry, for example, non-European ancestry (68); frailty (45) and older age (55–60, 68); genetic predisposition (68, 104–107), including gene variants at 3p21.31 (68, 107) and variants involved in immune signaling [e.g., TLR7 (105) and interferon (106)]; male sex (68); and current or recent pregnancy (61). Equally, past infection with other coronaviruses (84, 85), whether an individual has received monoclonal antibodies (108), and exposure history or vaccination status (type, provision of boosters, or intervals) (35, 66, 70–83) are also relevant. Comorbidities similarly affect the immune response, such as: whether an individual has a history of malignancy (66) or has received recent chemotherapy for cancer (63); has disorders of lipid metabolism (67); is a transplant recipient (62); has uncontrolled HIV (65); has hypertension (67); is obese (67, 68); or takes immunosuppressants (64). Other relevant variables include air pollution (45), microbiota composition (45), presence of co-infections (45), and socioeconomic status (45, 68).

In summary, viral and host elements modify the risk of reinfection or development of severe COVID-19 in various manners (Figure 1).

## A composite CoR: antibody dynamics, serology in practice and challenges, and expert recommendations

The antibody component of a composite CoR should be developed under defined conditions. To provide insight into these conditions, an understanding of antibody dynamics is required.

### SARS-CoV-2 antibody dynamics

Natural infection with SARS-CoV-2 elicits a diversity of antibodies including those targeting S and nucleocapsid (N) antigens (75, 109) and the development of anti-RBD IgG antibodies is associated with improved patient survival (110). A detailed systematic review of 66 studies investigated antibody responses (111). Collectively, the evidence supports the induction of IgM production in the acute phase of natural infection (peak prevalence: 20 days) followed by IgA (peak prevalence: 23 days), IgG (peak prevalence: 25 days), and nAbs (peak prevalence: 31 days) after symptom onset (111).

Serum IgG has the longest half-life compared with the relatively transient IgA or IgM (112). A longitudinal analysis of 4,558 individuals, measuring total anti-N antibodies, revealed that, while total antibodies begin to decline after 90–100 days, they may persist for over 500 days after natural infection (113). Specifically measuring nAb via plaque reduction neutralization test (PRNT) shows that infection yields a robust nAb response in most individuals (86). Some studies report that anti-S antibodies show greater persistence than anti-N antibodies (114, 115).

Dramatic inductions of anti-S or anti-RBD IgG antibodies is indicative of vaccination (75, 116, 117). Primary vaccination by some vaccines [but not all (118)], or boosters generates high nAb titers (117, 119, 120) or neutralizing responses (116). Notably, nAbs wane over time (35) with a half-life of 108 days (100)—although the level of decay may be assay or variant dependent (119) – and multiple clinical factors affect the duration of neutralization responses after primary vaccination (66) (see also Figure 1).

### Anti-SARS-CoV-2 antibody testing

#### Commercial high-throughput immunoassays

Numerous immunoassays for the detection of antibodies against SARS-CoV-2 are available, differing in the immunoglobulin class detected, target viral antigen, format, and output [qualitative, (semi)-quantitative] [reviewed in detail (121, 122)].

Head-to-head comparisons from the pre-Omicron era reveal variable levels of performance between the assays (123–127), caused by numerous technical factors including assay methodology, format and antibodies used, timing of testing, and the targeted viral antigen. Comparison studies show that sensitivity for detecting prior infection by different serologic assays changes over time (128). Commercial assays developed early during the pandemic are based on ancestral/wild-type antigens. Subsequently, there is potential for differential performance in the Omicron-era: in particular, S- and RBD-specific immunoassays have shown significantly reduced performance (129–131), and decreased comparability of quantitative results (132).

Most common commercial immunoassays detect both binding and nAbs without differentiating between them, however certain assays measuring IgG or total antibodies correlate well with neutralizing capacity (28, 97, 133–139), acting as surrogates of neutralization. Cell-based virus neutralization tests can be used to measure neutralizing capability, but these are typically not readily available in clinical laboratories due to inherent test performance challenges associated with their methodology (including the need for biosafety level 3 containment for live-virus neutralization assays), time and cost (140).

##### Expert recommendations

Mature immune responses are dominated by IgG. Serologic assays that measure IgG or total antibodies (if skewed toward IgG) that correlate with neutralizing activity and focus on anti-RBD should be used for the serologic component of a composite CoR; anti-N antibodies are unlikely to be neutralizing as the N protein is located within the viral envelope (75).

Assays should be adapted for accurate measurement of the modified antigen, if applicable. However, frequent adaptation of assays is unlikely if several variants are circulating in parallel and due to regulatory requirements for assays. Therefore, studies are needed to determine assay applicability in the present conditions, especially since RBD mutations frequently occur and recombinant versions of RBD or S are commonly used in immunoassays (122). Accordingly, the upper and lower thresholds of any CoR may need modification.

External ring trials show poor comparability of assays from different manufacturers (141, 142) and there are significant challenges with the current binding antibody units (BAU) standardization, due to multiple factors, including different assay methods, antibody class(es) detected and target antigen used. Of note, BAU reference materials were derived from UK convalescent individuals infected in 2020 (143) (pre-Omicron), and there are vastly different BAU standardized values (144). While new reference materials include VOCs, they still contain antibodies derived during the pre-Omicron era (145). Antibody measurements should be harmonized across assays from different manufacturers, irrespective of the different epitopes utilized, to reduce variability. To support this, there is an urgent need for external quality assessment, production of robust traceable certified reference materials, standards for different variants, and improved documentation of the methods on laboratory reports. Age-specific normalization of reference intervals in defined groups, by means of z-log transformation and documentation in antibody passes, may further improve the comparability of assays. Stakeholders should agree on minimum performance-based criteria to develop the gold standard for CoR, allowing validation of secondary assays.

Finally, systemic cellular assays could provide a comprehensive profile of the immune response, especially in immunocompromised and susceptible individuals who are not able to mount a robust antibody response. Currently, they lack scientific evidence and their use in clinical practice still remains uncertain.

#### Sample matrices

Systemic anti-SARS-CoV-2 antibody testing can be performed on blood, plasma/serum, or dried blood spots (DBS) (122, 146–148). An advantage of whole blood or DBS collection is the ease in obtaining the sample. While many methodologies focus on systemic testing, infection with SARS-CoV-2 or vaccination against COVID-19 induces mucosal antibodies (149, 150), thus secretions such as saliva offer another possibility. Antibody dynamics will differ depending on the material in question (151), and sample types are subject to specific idiosyncrasies, such as additional pre-processing, that need to be accounted for (152). Of note, the collection protocol (passive drool versus swab-stimulated saliva, for instance) can influence the antibody yield (153). Currently secretion-based testing is less suitable for a composite CoR as performance is variable (154).

##### Expert recommendations

A composite CoR will likely be sample matrix-specific. Our preference is for plasma/serum, as this sample matrix has the largest evidence base, shows the least variability, experiences less interference than whole blood, and is consistent with CoRs established for other infectious diseases. DBS would be also possible, but variability is high, and few laboratories have an established workflow.

#### Serologic testing formats

Formats include high-throughput automated enzyme immunoassay/ electrochemiluminescence immunoassay/enzyme-linked immunosorbent assay (certified and used in central laboratories and hospitals), point-of-care (POC) testing (used in emergencies and outpatients setting), and direct-to-consumer testing (at-home use with online services). POC testing is gaining in popularity, but methodological variation is higher (155) and any method that relies upon sampling from untrained individuals is less reliable for (semi)quantitative measurements (156).

##### Expert recommendations

We recommend automated assays that are approved by location-specific regulatory agencies and performed in certified and centralized laboratories. Home sampling/DBS would contribute to a reduction in clinician workload, particularly in high-density residential facilities, but methods are not yet sufficiently robust. Currently, there is no clear benefit in POC testing as urgent results are not critical.

#### Frequency of sampling and optimal time point

Considering antibody dynamics, several important questions arise: what is the optimal time point for measurement; would the timing differ depending on the vaccine schedule, and/or the presence of previous infection of a specified severity; should antibody levels be measured once or serially? While single values can be plotted into modeled curves showing decrease rates over time, serial measurements could further refine the composite CoR. Only individuals with symptomatic disease or vaccination are known to stabilize the curve—infections that are sufficiently mild to lack detection will impact the composite CoR model.

##### Expert recommendations

As most individuals have experienced infection or vaccination, and titers are generally high and more stable than with single exposures, sampling should be performed annually or less. Serologic evaluation should be conducted more frequently in the older adult or immunocompromised than the general population (time interval to be defined), depending on any underlying disease and/or treatment.

## Discussion

A composite CoR would be helpful particularly for high-risk groups, such as solid organ transplant recipients (157), and those in occupations with high risk of exposure to SARS-CoV-2. However, whether a composite CoR would operate at the individual or population level is yet uncertain.

For health policymakers, a composite CoR could be useful for: (1) predicting the durability of protection, supporting serosurveys to determine the protection levels of individuals and populations; (2) aiding decision-making with regard to monitoring vaccination efficacy and identifying individuals who would benefit from booster vaccinations; (3) evaluating the need for extra protection of vulnerable communities in the face of new variants with low cross protection and less efficacious vaccines; (4) licensing new vaccines; and (5) developing clear immunologic vaccine trial endpoints.

A previous systematic review by Perry and colleagues found mixed evidence for a serologic CoP, with the lack of standardization between laboratory methodology, differing assay targets and sampling time points, and the lack of information on the SARS-CoV-2 variant confounding interpretation (158). We have highlighted various parameters that should be controlled for in any measure of risk, some of which will be challenging to obtain (such as host genetics). Comparing different protection studies is also difficult as infectious pressure in the observation time period is often uncertain as, in reality, community data are incomplete and the number of oligosymptomatic infections is unclear. Of course, individual responses to infection and vaccination with regards to antibody production will make long-term assessment difficult, intrinsic risk will vary by age and protection will not be linear (139, 159). To ensure an acceptable level of accuracy, it will also be important to assess the composite CoR in geographic settings where extrinsic environmental factors, host genetic backgrounds, and circulating variants contribute to the overall effect on the immune response. All the variables previously described need to be thought of in the general context of laboratory diagnostics, paying attention to sensitivity, specificity, positive/negative predictive value, reliability, precision, dilution, linearity, robustness, stability, preanalytics, scalability (automation), cost-efficiency, *In Vitro* Diagnostic Regulation certification, and the use of qualified standard and control materials. Laboratory quality is essential for meaningful follow-up of quantitative antibody levels.

While the development of a composite CoR is a sizeable undertaking, steps can be taken to address this need. Studies need to adapt to the requirements of new variants, controlling for patient settings (vaccination types, earlier infections), and levels of disease severity. The emergence of VOCs means that a CoR will undoubtedly be variant-specific and the timing of infections and vaccination, how variants impact disease severity, antibody kinetics, and assay reactivity, must be respected. Frequently revisiting the data would be helpful as overall epidemiology changes; since almost all epidemiologic population-based studies have ended, background data is increasingly difficult to acquire, and this must be reversed. While serologic testing has retreated from the political agenda and public interest, we have an obligation to broaden the scientific knowledge base, and collect data to inform public health authorities, given that COVID-19 still causes a significant number of deaths and there is a considerable population of those with post-acute sequelae of SARS-CoV-2 infection [long COVID; (160)].

A composite CoR will differ depending on the clinical endpoint (26). Definitions of symptomatic or severe disease are often not consistent across studies (100). Clinical outcomes must be precisely defined: an evaluation of the primary endpoints of 19 clinical trials for severe COVID-19 revealed the complexity of this task, reporting 12 different primary endpoints (161). In addition, the ideal timeframe for predictive ability is yet to be determined.

While we support the development of a composite CoR and serologic testing by high- quality controlled assays, viruses such as influenza have significant strain variation and similar disease severity, so the importance of a composite CoR for SARS-CoV-2 should be judged against other pathogens of interest. Assessment of cost-effectiveness will likely inform upon the need for a composite CoR.

## Data availability statement

The original contributions presented in the study are included in the article/supplementary material, further inquiries can be directed to the corresponding author.

## Author contributions

SH: Conceptualization, Writing – original draft, Writing – review & editing. CD: Conceptualization, Writing – review & editing. JI: Conceptualization, Writing – review & editing. ET: Conceptualization, Writing – review & editing. AW: Conceptualization, Writing – original draft, Writing – review & editing.
